# Paul Eelen: Reflections on Life and Work

**DOI:** 10.5334/pb.468

**Published:** 2018-07-26

**Authors:** Dirk Hermans, Frank Baeyens, Tom Beckers, Geert Crombez, Jan De Houwer, Agnes Moors, Filip Raes, Omer Van den Bergh, Bram Vervliet

**Affiliations:** 1 Faculty of Psychology and Educational Sciences, KU Leuven, BE; 2 Faculty of Psychology and Educational Sciences, Ghent University, BE

**Keywords:** conditioning, behavior therapy, Paul Eelen, legacy

## Abstract

This manuscript is part of a special issue to commemorate professor Paul Eelen, who passed away on August 21, 2016. Paul was a clinically oriented scientist, for whom learning principles (Pavlovian or operant) were more than salivary responses and lever presses. His expertise in learning psychology and his enthusiasm to translate this knowledge to clinical practice inspired many inside and outside academia. Several of his original writings were in the Dutch language. Instead of editing a special issue with contributions of colleagues and friends, we decided to translate a selection of his manuscripts to English to allow wide access to his original insights and opinions. Even though the manuscripts were written more than two decades ago, their content is surprisingly contemporary. This introductory article presents a reflection on Paul’s career and legacy and introduces the selected manuscripts that are part of this special issue.

On August 21, 2016, prof. dr. em. Paul Eelen passed away. Paul was one of the prominent Belgian psychological scientists of his time. To commemorate his legacy, we compiled this special issue for Psychologica Belgica. Rather than inviting an international forum of Paul’s colleagues to write contributions for this issue, we decided to translate a selection of his Dutch publications to English and make them accessible for a wider audience. Even though the majority of these contributions is more than two decades old, the ideas that they transpire are still highly contemporary. The editors of this special issue and authors of this introductory article are all ‘academic children’ of Paul and had the pleasure to have experienced him as a supervisor and mentor. In this article we reflect on Paul Eelen’s career and legacy and introduce the selected readings that are part of this special issue.

## Paul Eelen’s career

Paul Eelen was born in Berchem (Belgium) on May 23 1941. Paul’s father was a renowned engineer and director of major constructions in the harbour of Antwerp, which he combined with a part-time professorship at the university of Leuven. During secondary school, Paul did classic studies at the Sint-Stanislas College (Berchem), which he completed in 1958. In line with a tradition among many Flemish-Belgian families to have one of the older sons study for priest, Paul entered the seminary of Mechelen immediately thereafter. Later, he started his academic education at KU Leuven (then: Catholic University of Leuven) to study theology, part of which he completed at Strasbourg (France). He obtained his degree at the Faculty of Theology in 1965. Even though Paul was already ordained as a catholic priest (in 1961), he soon left priesthood. When he informed the bishop about his wish, he was asked to take this decision into consideration for one year. During that year Paul was allowed to start his studies in psychology. He combined the first two years of psychology with the completion of his master’s thesis in theology. Even though he also considered a medical career, the choice for psychology was a firmly positive one. Paul was interested in the mechanisms that drive human behaviour, particularly in the domain of clinical psychology. He obtained his master’s degree in psychology in 1969 in the newly established Faculty of Psychology and Educational Sciences. In December 1967 the ‘Institute for Applied Psychology and Pedagogy’ was transformed into the new faculty. The first dean of this new Faculty was professor Joseph Nuttin Sr., who supervised Paul’s master’s thesis, which was titled: ‘*The contribution of Charlotte Bühler in the study of motivation*’. This monography of almost 250 pages started with an elaborate section on the life and work of this German psychologist; a section that was inspired by regular postal mail correspondence between Paul and this author. Even though she was known for her developmental work, Paul analysed her impact on the domain of motivational psychology, which was one of the core topics of his own supervisor. The thesis also contained an empirical study that compared three methods of motivational assessment, including Bühler’s ‘Life Goals Inventory’ and two methods that were developed in Leuven.

Soon after the completion of his master’s degree in psychology, Paul Eelen embarked on a PhD project, again under the supervision of prof. Nuttin. The Faculty was then situated in a formally Dutch-speaking university after the split in a Dutch and a French speaking university in 1968 and the move of the latter to the newly built campus at Louvain-La-Neuve for which the construction started in January 1969. Even though Paul’s work was conducted in Leuven, his doctoral and postdoctoral years were funded by the National Foundation for Scientific Research (NFWO). Between October 1969 and September 1970, he first started as an ‘intern’ of the NFWO (‘navorsingsstagiair’; a category that does no longer exist) and was ‘aspirant FWO’ from October 1970 to September 1974. His PhD was successfully defended on January 17^th^ 1974. After completion of his doctoral dissertation, Paul continued his career at the NFWO, first as an ‘aangesteld navorser’ (until 1978) and then as ‘bevoegdverklaard navorser’ (until 1984).

Even though his doctoral work with Prof. Nuttin Sr., who was a world-leading scientist at that time, has stimulated Paul’s love for the experimental methodology, particularly his post-doctoral years gave form to his passion for the combination and integration of basic psychological science and clinical psychology. During his postdoctoral research, Paul and his family (his wife Rita Beckx and their three sons Jan, Bert and Dirk) moved to the United States for two lengthy periods. In 1976–1977, a research visit was conducted at the University of Pennsylvania, Philadelphia, where Paul collaborated with prof. Martin Seligman and prof. Richard Solomon. Martin Seligman was renowned for his work on learned helplessness and its implications for the understanding of depression. Richard Solomon was known for his work on avoidance learning and the opponent-process theory of emotion. The Eelen family was warmly welcomed and were allowed to stay with the Seligmans during their first weeks in the States, while looking for their own house. A second lengthy stay in the States brought Paul to Temple University (Philadelphia), where he completed his therapy training under the supervision of Joseph Wolpe (1978), who was a real pioneer in behaviour therapy. In a letter to his uncle Paul describes how interesting and rewarding his work with patients was at the Department of Psychiatry. The two research stays have been crucial in further fuelling Paul’s interest in applying the experimental study of human behaviour (and in particular learning psychology) to clinical phenomena.

Upon his return, Paul started teaching at his home university and became part-time assistant professor in 1979. In 1984 he was appointed as full-time assistant professor and moved swiftly through the ranks of professorship: professor (hoogleraar) in 1987, and full professor (gewoon hoogleraar) in 1990. In 1979, Omer Van den Bergh joined Paul as his first doctoral student. This marked the start of his own research center, which he coined ‘*Center for the Psychology of Learning and Behaviour Therapy*’. Note that this name reflects his combined interest in basic experimental psychology and the clinical domain.

Within the field of behaviour therapy, that was then moving from an underdog position to a well-respected approach, Paul soon became a front-runner in Belgium and the Netherlands. He was an often-invited keynote speaker at clinical meetings, was president of the *Vlaamse Vereniging voor Gedragstherapie (Flemish Association for Behaviour Therapy)*, board member of the Dutch-Flemish journal *Gedragstherapie (Behaviour Therapy)*, and later author of a leading introductory book on behaviour therapy (Orlemans, Eelen & Hermans, 1996; Hermans, Eelen & Orlemans, 2007). As a newcomer in the field, Paul organised (together with Ovide Fontaine) the 1984 edition of the ‘*Annual Congress of the European Association of Behaviour Therapy*’ in Brussels (Eelen & Fontaine, 1986). In line with his firm belief in the necessity of grounding clinical practice in basic science, each of the three days of the conference was organised along one of the three core themes: psychophysiology, social psychology and cognitive psychology. Unlike other similar clinical conferences, keynote speakers included famous basic scientists: Peter Lang, Terrence Wilson and Gordon Bower. Later, Paul incorporated the existing training in cognitive behaviour therapy within the academic setting and became a supervisor and mentor for many practitioners. It was not with large studies that Paul pushed the clinical field forward, but by a unique combination of sharp intelligence, enthusing passion, deep knowledge of the literature, bold statements, and a clear view on how clinical practice should always be rooted in basic science. Paul conveyed his ideas in numerous contributions for (local) clinical journals, which were often science-based ‘sermons’ in pursuit of his ideals. The two papers in this special issue on ‘*The therapist as Conditioned Stimulus*’ and ‘*The broken Achilles heel of behavior therapy: A couple of reflections on the function analysis*’ are examples of such manuscripts. He was also invited internationally to give addresses on these topics.

Paul enjoyed lecturing and teaching. During his career he had a full teaching load which included general introductory psychological courses and more advanced teaching on the ‘Psychology of Learning’ and ‘Behaviour Therapy’. But foremost he loved teaching ‘The History of Psychology’. Pavlov’s quote that could be found on Paul’s office door typified him very well: ‘*If you want new ideas, read old books*’. And, in spite of the fact that Paul was a charismatic teacher – sometimes a bit the prototype of the absent-minded professor – he remained nervous for every class; an emotion that never extinguished despite tons of positive reinforcement. On more than one occasion, we observed him driving to the conference location with a car full of books, missing most of the conference while preparing his keynote in his hotel room, to finally present a lecture that we actually had heard before in some form, but that was still refreshing and moved the audience. Paul’s good friend and colleague prof. Tom Borkovec wrote about one of these lectures: “*The most memorable for me was his keynote speech at the European Association of Behaviour and Cognitive Therapies in Maastricht in September 2002. The title of his talk was ‘A conditioning perspective on anxiety disorders’. There were insufficient seats in the large room because of the overflowing attendance, and at the end of his talk, he received a standing ovation that lasted longer than I have ever witnessed in my 34 years in my profession*”.

By the end of the 1980’s, Paul’s research group started to grow and flourish. Amidst a university that was steadily transforming into a research-heavy academic setting, Paul was a true leader. He attracted excellent young researchers, who he allowed to function in an atmosphere of true academic freedom. He started organizing a yearly retreat for a full week to elaborately discuss ongoing studies and new research ideas. With himself and Rita doing the exquisite cooking, he was able to establish a unique family atmosphere among the collaborators realizing what he considered the hallmark of doing research, namely that it should be fun to do. Inspired by his insights and ideals, Paul’s group was gaining strong international visibility and reputation. While the rest of Europe was riding on the waves of cognitive psychology, Paul kept a steady behavioural research program with a strong focus on human learning. His persistence turned out to be visionary. Around the turn of the century, conditioning regained international interest and the expertise that Paul had been building over the years in this domain propelled his group to a world-leading status. He applied for and obtained large-scale research grants. Papers were published, special issues edited, special interest meetings organized, and dozens of studies conducted. During the 1990s, Paul was an active member of the National Science Foundation (now FWO-Flanders), and in 1992 he co-organized the 25^th^ International Congress of Psychology in Brussels (Bertelson, Eelen, & d’Ydewalle, 1994). With several thousand attendants from over 70 countries this proved to be an immense job. Paul’s contributions to the field were honoured in many ways. He was an honorary member of the associations for cognitive behaviour therapy in Belgium and the Netherlands, and in 1995–1996 he received the Francqui Chair by invitation of UCLouvain. The title of a manuscript by Marcel van den Hout and colleagues ‘*Lang leve Leuven!*’ (‘*Long live Leuven*’; van den Hout, de Jong & Arntz, 1998) reflects the impact that Paul had on the field.

**Figure:**
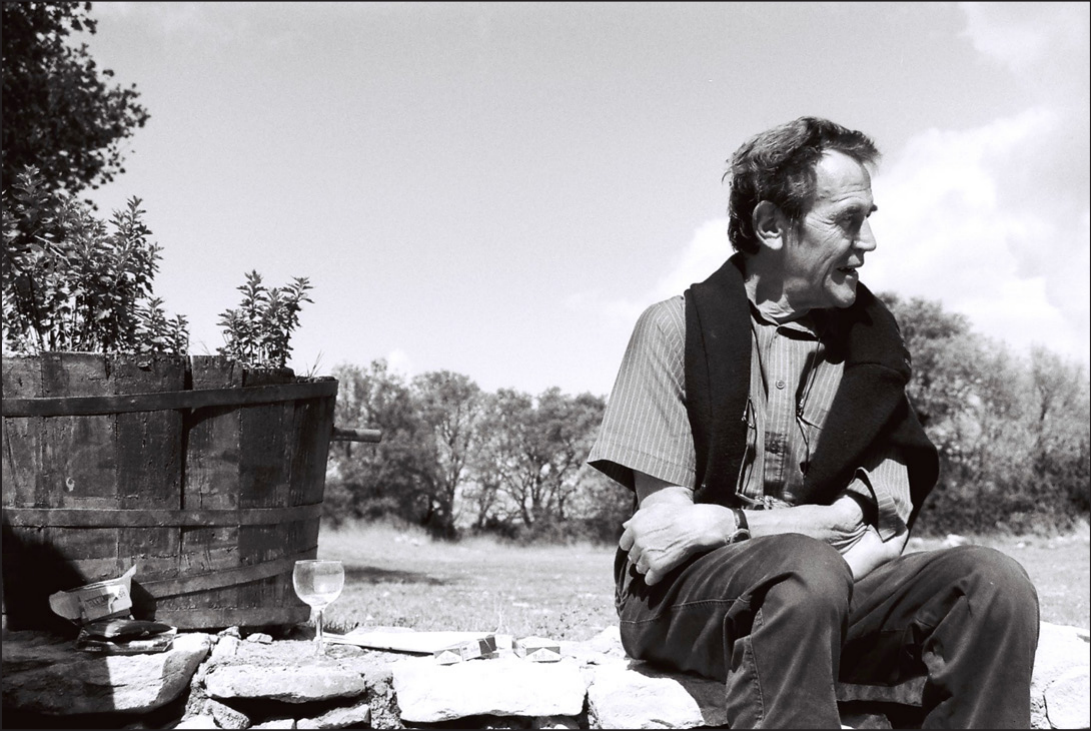
Paul Eelen in September 2005 (picture: Tom Beckers).

On June 9 2006, Paul Eelen celebrated his retirement in the presence of family, friends and colleagues. He shied away from a traditional academic ceremony and left the organisation of a more personal and intimate festivity to his collaborators. He did, however, express one explicit wish, which was to invite Joost Zweegers (Novastar) – a celebrated musician and friend of the family – to fly over and play one single song: ‘*The best is yet to come*’. And indeed, leaving behind a legacy of dozens of chapters and articles, solid scientific knowledge and a team that would further his work, from then on Paul invested his warmth and generosity unremittingly to his family and friends.

## Paul Eelen’s legacy

Paul Eelen’s direct influence was considerable, on the academic but perhaps even more so on the clinical scene. As indicated above, upon his return from the States in 1979, and inspired by his training with Joseph Wolpe and interactions with Tom Borkovec, he put his full weight behind the emerging field of behavior therapy in Flanders, providing it with a strong academic embedding and solid organization.

He would remain massively influential on the field throughout his academic career, organizing and supervising the training of generations of behavior therapists, writing several editions of the canonical Dutch-language introductory behavior therapy handbook (with Hans Orlemans and Dirk Hermans), editing a long-running series of specialist behavior therapy chapters (with Hans Orlemans and Willem Haaijman), and acting as the public academic conscience of behavior therapy and a guardian of its rigor in the low countries.

Anecdotally, the fact that to this day, the Flemish association of behavior therapists is called “Vlaamse Vereniging voor Gedragstherapie” [Flemish Association for Behavior Therapy, VVGT] and not “Vlaamse Vereniging voor Cognitieve en Gedragstherapie” [Flemish Association for Cognitive and Behavior Therapy], unlike its Dutch counterpart (“Vereniging voor Gedrags- en Cognitieve Therapieën” [Association for Behavior and Cognitive Therapies, VGCt]), is a clear indication of Paul’s heritage. Surely there are pragmatic reasons for practitioners to adopt the CBT label that has come to represent the amalgamation of approaches that have emerged as behavior therapy, cognitive therapy, third-wave CBT and beyond, reasons that eventually persuaded the Dutch to rebrand the VGT into VGCt against Paul’s better advice. As an academic, however, Paul was convinced that solid scientific thinking ought to trump pragmatics, and he argued fiercely against what he called the “category mistake” of treating behavior and cognition on the same footing. After all, he maintained, behavior refers to an observable, whereas cognitions are necessarily unobservable. Cognitions can be invoked as explanans to help understand behavior as an explanandum, but cognition and behavior should not be set in opposition as if they refer to the same level of analysis and as if behavior therapy and cognitive therapy would be fundamentally different things. To state it differently, in Paul’s view, verbal behavior should not simply be equated with cognition. Verbal behavior to him was just another instance of behavior, and the mere fact that a therapy focuses mostly on the patient’s verbal behavior would not make it any less an instance of behavior therapy in his eyes.

Paul’s enormous contributions to the development of behavior therapy in the low countries would eventually earn him honorary memberships of both the VVGT and the VGCt, a unique accomplishment that he shared only with Hans Orlemans.

Paul Eelen’s academic influence was considerable as well. He was a sharp thinker, who profoundly influenced generations of students and collaborators. He sole-handedly put learning theory on the research agenda in Flanders. Some of his writings on the topic, where he argued against the idea that conditioning would be nothing more than “the psychology of spit and twitches”, predate the work of internationally acclaimed scholars on the topic (see below). However, the direct academic and clinical impact of Paul’s work was mostly restricted to the local (Flemish and Dutch) level, in part due to his hesitance to publish in English, a hesitance that was inspired not by insufficient mastery of the English language but by an unwarranted modesty about the originality and importance of his ideas. We hope that the present volume may contribute to the retrospective rectification of that situation.

Nonetheless, his indirect influence clearly transcended the local level and continues to do so to this day. Paul created a legacy that has an enduring impact on academic psychology and clinical practice, in large part thanks to his exceptional ability to attract and nurture an ever-growing group of talented PhD students and postdocs, many of whom would go on to secure faculty positions at KU Leuven and elsewhere and gain great international visibility and impact. Through those former students, who would in many cases become key scholars in their respective areas, Paul had an indirect but enormous international influence on a diversity of fields, including the psychology of learning and memory (e.g., through people like Frank Baeyens, Jan De Houwer, Tom Beckers, Bram Vervliet), health psychology (Omer Van den Bergh, Geert Crombez), social psychology and social cognition (Jan De Houwer, Adriaan Spruyt, Hilde Hendrickx), emotion science (Agnes Moors), experimental psychopathology and clinical psychology (Dirk Hermans, Debora Vansteenwegen, Filip Raes), and others. In all those fields, Paul and/or his students have indeed made widely acknowledged and transformational contributions that are of continuing influence up to this day, which can be deemed truly exceptional.

One may wonder why Paul’s progeny was so successful. No doubt was he lucky to attract excellent students, thanks to his keen eye for talent and his charisma that readily lured those talents to his lab. But talent is replete in academia. What set Paul apart was his exceptional gift for creating an academic environment in which such talent could thrive. More than individual ability and accomplishment, he believed in the power of collaboration and the collision of ideas. He established a lab culture that put a great emphasis on cooperation and exchange, installing weekly lab meetings long before they became a universal fashion. At those lab meetings, everyone’s research designs were critically evaluated and their conceptual and methodological underpinnings were scrutinized respectfully but also relentlessly and without mercy, with Paul providing the historical and philosophical backbone derived from his vast knowledge of not only the empirical literature on associative learning and conditioning but also the history and prehistory of psychology. Despite this enormous philosophical background and empirical knowledge (and in spite of the directive reputation of the psychotherapeutic orientation that he promoted so fiercely), Paul was moreover entirely non-directive in the guidance of his students, who were basically left to study whatever they saw fit (which explains in part why they would go on to make a mark on such a diversity of fields in psychology).

It is remarkable that the thematic freedom that Paul’s students enjoyed, and the diversity in their research topics that it spawned, never stood in the way of lab members’ mutual interest, intellectual investment and collaboration. Paul’s secret weapon to this effect was probably that he fully recognized the importance of the social dynamics of a research group for its scientific fertility. In addition to the weekly lab meetings, he installed a tradition of yearly retreats, where in addition to heated scientific discussions, there was room for fun and games (and drinking – somehow the literature on the adverse effects of alcohol on learning and memory seemed to have been lost on Paul). At those retreats, as in the lab, dealings were informal and non-hierarchical, the input of freshly arrived students was valued as much as that of established postdocs, and the cooking was done by Paul himself, as a benevolent pater familias.

And while Paul never managed to reach great international impact directly, he strongly recognized the importance of international exposure and networking for his students. He encouraged them to go and spend time in prominent international labs in the States and elsewhere and to publish in prime international specialty journals, and he also actively brought the international research community to the lab, by organizing (or having his students organize) international expert meetings that would gather the absolute world experts on a given topic in a secluded house in the Ardennes for a couple of days – he himself again taking a humble role as cook for those international guests. Those expert meetings not only provided unique exposure to the best international scholars in a given topic for his students, but in turn yielded great international visibility for those students as well, as they often resulted in enduring international contacts and in special issues or book volumes on the topic of the meeting (co-)edited by his students. And while his students thrived, Paul himself thrived in the knowledge that his academic and personal generosity helped to make that possible.

## Paul Eelen: selected readings

We have chosen five contributions of Paul to form the body of this special issue: three manuscripts, a talk, and an interview with Paul. We believe this selection nicely conveys several of Paul’s most inspiring ideas and some of his pet scientific topics. Paul mostly published in Dutch, and so we are really pleased that the English-speaking community can now also be inspired by Paul’s ideas through these translations. What also becomes clear from all five contributions, is that Paul’s ideas and considerations are as relevant today as they were at the time. In what now follows, we offer a brief introduction to the five contributions and we highlight what we see as the core or main message of each contribution.

The first contribution is a manuscript entitled “Classical conditioning: Classical yet modern”. Paul wrote it in 1980 for the Festschrift that was published in honour of the retirement of his mentor Prof. Nuttin Sr. It was written at a time that conditioning research was considered by many cognitive psychologists as the final stronghold of a dying breed of behaviourist researchers. Paul pointed out that this misconception resulted from an overly restrictive view on conditioning as a non-cognitive mechanism via which S-R associations are automatically formed between conditioned stimuli and unconditioned responses that happen to co-occur. Interestingly, the same message was several years later put forward by Rescorla (1988) in his famous *American Psychologist* paper. Unfortunately, Paul’s as well as Rescorla’s paper are still highly relevant today for the many (neuro)psychologists who still think of conditioning in terms of the automatic formation of S-R associations. To illustrate the complexity of conditioning, Paul reviewed the then recent literature on conditioning, including studies demonstrating the role of contingency (rather than mere co-occurrence) in shaping conditioned responses, cutting-edges on studies on cue competition, as well as research on taste aversion. Although it is interesting to see how he skilfully summarised this literature, the truly original part of the paper lies in the final section in which Paul explored the link between conditioning and causal attribution as it was studied by social psychologists such as Kelley (1967). He pointed at the many similarities between both phenomena and argued that there is merit in entertaining the hypothesis that rats in conditioning experiments behave “as if” they are making causal attributions, a proposal that again forbade the conclusion of Rescorla (1988, p. 154) who favoured “an analogy between animals showing Pavlovian conditioning and scientists identifying the cause of a phenomenon.” Current research shows that Paul’s ideas about the link between conditioning and attribution are as sharp and inspiring as they were 40 years ago (e.g., Blaisdell et al., 2006; Beckers et al., 2006).

Also in 1980, Paul gave a talk on behaviour therapy and behaviour modification for the alumni of the Faculty of Psychology and Educational Sciences of KU Leuven, which is the second contribution in this issue. Knowing that Paul did not use slides and typically relied only on a few bullet points that he listed for himself, the talk illustrates that Paul was an excellent storyteller: He used historical anecdotes associated with landmark publications and events to contextualise the emergence of behaviour therapy and the struggle of this “new kid on the block” to gain scientific respect in a time that was dominated by the psychodynamic approach. Paul used this narrative style mixing anecdotal stories, simple examples illustrating fundamental questions, and a thorough knowledge of the literature, to critically elaborate on four characteristics that would define behaviour therapy according to Kazdin (1978). At the end, he apologised for playing too much the devil’s advocate in questioning each of these widely accepted characteristics. For example, he asked what is meant by the assumption that abnormal behaviour is learned. And whether it is true that behaviour therapy is putting findings of experimental psychology into practice, or in nowadays terms, whether behaviour therapy is really as evidence-based as it claims to be. He also questioned what should be considered “behaviour” when it is claimed that behaviour therapy is focusing on changing behaviour. The talk eloquently illustrates why it remains so difficult to establish that behaviour change took place as a result of the therapist’s interventions, and why it is even more difficult to determine the mechanisms underlying the change. This is yet another set of insights that have stood the test of time.

The third contribution was published as a chapter of a book published at the occasion of the retirement and farewell of Prof. Jan Rombauts, founding father of the “Centre for Client-centred Therapy” at KU Leuven. In line with the title of the book, namely “De relatie in therapie” (The relation(ship) in therapy), and amidst chapters on “focusing”, “the working relationship in client-centred psychotherapy”, “countertransference”, or “the helping relationship in the context of experiential training theory”, Paul came up with a piece with the slightly provocative title “The therapist as conditioned stimulus” (De therapeut als geconditioneerde stimulus). The chapter (co-authored with partners in crime Eric Depreeuw & Omer Van Den Bergh) is vintage Paul Eelen, both in style and content: well-structured and characterised by tight argumentation, but leaving room for the occasional meandering or associative digression; didactical foremost, preaching at places; on-topic most of the time, but grasping the opportunity to reiterate his scientific credo and firm beliefs about the core principles of therapy wherever possible.

In the first part of the text, he explains “why behaviour therapists remain *slightly hesitant* (italics added) to accept the basic philosophy underlying client-centred therapy”, and then goes full force, characterising the scientific literature on client-centred therapy as (1) lacking adequate operationalisations of the key concepts, (2) lacking reference to more general psychological concepts and scientific theory, and (3) demonstrating an unwarranted disconnect between the concepts of “experience” and “behaviour”. Bam! So far for the prospect of therapeutic ecumenicalism: no way to bridge the gap. Developing his arguments, this section also contains some true gems, for example the harsh but fair judgement about Carl Rogers’ classical paper *The Necessary and Sufficient Conditions of Therapeutic Personality Change*: “Had the same article been written by a novice therapist, it would have barely been considered suitable for publication and it certainly would not have had the same influence”, or still: “what Rogers and many researchers in client-centred therapy later labelled as “therapeutic factors” are not factors but consequences of certain, insufficiently explained factors”.

The second, most substantial part of the text is devoted exclusively to the topic announced in the title of the chapter and covers an analysis of the therapist-client relationship from a learning psychological perspective. After an (admittedly a bit shallow) analysis of aspects of verbal and nonverbal therapeutic interactions from an operant perspective, ending in another classical quote (“We are manipulators after all! The analyst and the behaviour therapist do this explicitly; the client-centered therapists deny it, but they do it too.”), Paul delves into a crisp and clear analysis of therapeutic interactions from a Pavlovian learning perspective. Here he feels like a fish in the water, and starts with a scholarly discussion of the several different ways that stimuli can acquire (emotional) meaning through Pavlovian contingencies. Extrapolating from a variety of experimental observations on human and nonhuman animal Pavlovian learning, the novel and important insight that is developed here, is that especially *Pavlovian inhibitory learning* may be at the core of several phenomena that can be observed in the therapist – client interaction, both beneficial/desirable and potentially harmful/undesirable. In particular the high likelihood of the therapist becoming a *safety signal* is described as potentially hampering true therapeutic progress (that is, with respect to genuine extinction of the undesirable associations at the core of a problem), leading to the important insight that “putting emphasis on what happens outside rather than inside the therapy room” is quintessential for any effective (psycho)therapy.

As a fourth piece, we chose a manuscript of Paul from 1992 that he wrote together with Omer Van den Bergh for the clinical journal ‘Psychotherapeutisch Paspoort’, entitled ‘*The broken Achilles tendon of behaviour therapy: A couple of reflections on the function analysis*’. In this manuscript, Paul reflects on the crucial role of function analysis in behaviour therapy and points to the fact that this type of analysis is increasingly neglected. Functional analysis refers to the analysis of the factors that maintain (problematic) behaviour, in other words, a search for the function of this behaviour. Paul protested against the cookbook syndrome in which therapeutic interventions are chosen according to diagnostic labels based on topographic behavioural descriptions. Instead, he promoted an approach in which interventions are tailored to the functions of behaviour, which can vary from one person to the next. The debate on the problems of the traditional categorical approach in therapy is now more topical than ever. Recent attempts to overcome certain of its weaknesses led to alternatives such as the now-popular network approach. Although this approach has substantial promise in that it considers symptoms independent of diagnostic categories and can be tailored to the individual, the descriptions of the very symptoms remain topographic in essence. Therefore, we believe, together with Paul, that the function analysis deserves to be reinstated as the cornerstone of behaviour therapy and therapy altogether.

The final contribution is an interview with Paul for Veto – the student newspaper of KU Leuven – as part of a series on Science and Society. In this series, several scholars of the University were given the floor to freely elaborate on societal issues from their particular point of view, and Paul was selected to give his perspective on behavioural science. In the interview, he described the personal circumstances that led him to study psychology, and how learning psychology, and behavioural science more broadly, shaped his understanding of human normal and pathological behaviour. The interview is particularly relevant because it shows that Paul was a philosopher at heart. In colloquial language, Paul used simple examples from everyday life to briefly illustrate his view on complex issues such as the mind-body gap, the divide between natural and behavioural sciences, the boundaries of neuroscience, the importance and limitations of behavioural experiments, and the tension between determinism and the free will. Combined with short sketches of faculty life at that time, the interview demonstrates Paul’s deep conviction of what science is about: It is all about learning to think.
